# Bioanalytical Challenges due to Prior Checkpoint Inhibitor Exposure: Interference and Mitigation in Drug Concentration and Immunogenicity Assays

**DOI:** 10.1208/s12248-021-00643-4

**Published:** 2021-10-04

**Authors:** Andrew F. Dengler, Rachel Weiss, Tiffany Truong, Susan C. Irvin, Nidhi Gadhia, Mohamed Hassanein, Camille Georgaros, Jessica-Ann Taylor, Anne Paccaly, Giane Sumner, Matthew D. Andisik, Albert Torri, Michael A. Partridge

**Affiliations:** 1 Regeneron Pharmaceuticals, Bioanalytical Sciences, 777 Old Saw Mill River Rd, Tarrytown, New York 10591 USA; 2 Present Address: Pfizer, 401 N Middletown Rd, Pearl River, New York 10965 USA; 3 Regeneron Pharmaceuticals, Pharmacometrics (DSP), 777 Old Saw Mill River Rd, Tarrytown, New York 10591 USA

**Keywords:** clinical impact, drug concentration assay, immunogenicity, monoclonal antibody therapeutic, prior biologic exposure

## Abstract

Monoclonal antibodies (mAbs) are a leading class of biotherapeutics. In oncology, patients often fail on early lines of biologic therapy to a specific target. Some patients may then enroll in a new clinical trial with a mAb specific for the same target. Therefore, immunoassays designed to quantify the current mAb therapy or assess immunogenicity to the drug may be susceptible to cross-reactivity or interference with residual prior biologics. The impact of two approved anti-PD-1 mAbs, pembrolizumab and nivolumab, was tested in several immunoassays for cemiplimab, another approved anti-PD-1 mAb. The methods included a target-capture drug concentration assay, a bridging anti-drug antibody (ADA) assay and a competitive ligand-binding neutralizing antibody (NAb) assay. We also tested bioanalytical strategies to mitigate cross-reactivity or interference in these assays from other anti-PD-1 biologics. Both pembrolizumab and nivolumab cross-reacted in the cemiplimab drug concentration assay. This was mitigated by addition of antibodies specific to pembrolizumab or nivolumab. ADA specific for pembrolizumab and nivolumab did not interfere in the cemiplimab ADA assay. However, pembrolizumab and nivolumab generated a false-positive response in a target-capture NAb assay. Our results demonstrate that similar exogenous pre-existing anti-PD-1 mAbs (biotherapeutics) such as pembrolizumab and nivolumab are detected and accurately quantified in the cemiplimab drug concentration assay. However, once steady state is achieved for the new therapy, prior biologics would likely not be detected. Cross-reactivity and interference in immunoassays from previous treatment with class-specific biotherapeutic(s) pose significant bioanalytical challenges, especially in immuno-oncology.

**Graphical abstract:**

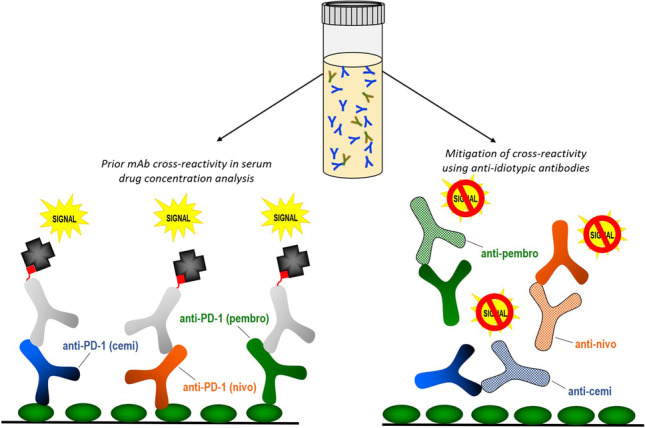

## INTRODUCTION

Biotherapeutics and especially monoclonal antibodies (mAbs) are one of the fastest growing segments of the pharmaceutical industry (1). This trend has accelerated with the approval of approximately 30 novel mAbs between 2017 and 2020 alone (https://www.fda.gov/drugs/new-drugs-fda-cders-new-molecular-entities-and-new-therapeutic-biological-products/novel-drug-approvals-2017,^2^).

Two therapeutic areas in particular have witnessed rapid growth in recent years: cancer immunotherapies, also referred to as immuno-oncology (IO), and immunology and inflammatory diseases (I&I, e.g., anti-TNF-α) (2, 3). This has resulted in an increasingly competitive and crowded treatment landscape. In some cases, hundreds of biotherapeutics engaging the same targets are under investigation in clinical studies (see Table I) (4, 5).

**Table I:** List of mAbs Approved or in Clinical Development for the Top 10 Targets in I&I and IO

Target	Approved	In clinical development	Total
EGFR	4	207	211
HER-2	8	186	194
PD-1	5	152	157
CD20	9	130	139
PD-L1	3	132	135
TNF-α	15	118	133
CD19	6	114	120
IL-6	3	48	51
CD38	2	26	28
IL-5	3	10	13

*EGFR* epidermal growth factor receptor, *HER-2* human epidermal growth factor receptor 2, *PD-1* programmed cell death protein 1, *PD-L1* programmed death ligand-1, *TNF-α* tumor necrosis factor α, *IL-6* interleukin 6, IL-5 interleukin 5

This redundancy is likely beneficial for both patients and health care providers as it provides multiple therapeutic options. However, it also increases the potential for patients to receive different biotherapeutics (approved or investigational) against the same target. Patients experiencing disease recurrence, non-responders, and those developing resistance may enroll in clinical trials involving related therapies; for example, patients may be anti-PD-1 experienced when enrolling in another anti-PD-1 study (6, 7). In the last few years, the Food and Drug Administration (FDA) and European Medicines Agency (EMA) have approved seven immune check point inhibitors (CPIs): one monoclonal antibody targeting the CTLA-4 pathway (ipilimumab), three targeting PD-L1 (atezolizumab, avelumab and durvalumab), and four targeting PD-1 (cemiplimab, dostarlimab, nivolumab, and pembrolizumab), for the treatment of patients with multiple cancer types (8–10).

In life-threatening diseases like cancer, patients failing to respond to one CPI may not be able to wait for clearance of the therapeutic before enrolling in a clinical trial with a treatment regimen consisting of a different CPI in combination with a novel experimental therapy (11). With the limited number of immunoassay formats commonly used in clinical trials to measure drug concentrations, anti-drug antibodies (ADA), and neutralizing antibodies (NAb), residual systemic drug that binds the same target may cross-react or interfere in these assays. For example, target-capture immunoassays that measure the concentration for one mAb therapeutic might be susceptible to cross-reactivity from different therapies directed to the same target. In cases where patients change to a new therapy of the same class before the prior therapy has been cleared, this may result in the detection of preceding therapeutics (6).

Bioanalysis of samples collected from patients treated with cemiplimab in two oncology trials revealed unexpectedly high concentrations of drug detected in baseline (pre-dose) samples in the target-capture cemiplimab drug concentration assay. The measurable drug concentrations of up to 95 µg/mL, similar to steady state cemiplimab concentrations (12–14), could not be explained by high background, matrix interference, analytical errors, or sample collection errors. The baseline samples with detectable drug were from patients enrolled in studies that allowed prior treatment with an anti-PD-1 biotherapeutic, including pembrolizumab and nivolumab.

Like cemiplimab, pembrolizumab, and nivolumab are both human IgG4 mAbs specific for PD-1 and are approved for a variety of oncology indications (15). Since the cemiplimab drug concentration assay uses PD-1 as the capture reagent, and a non-specific anti-IgG4 as the detection reagent, we investigated the potential for these two similar anti-PD-1 therapies to interfere with or cross-react in the cemiplimab drug concentration or immunogenicity assays (14, 16, 17).

Analysis of *in vitro* serum samples spiked with these therapies established that both pembrolizumab and nivolumab could be detected in the target-capture cemiplimab drug concentration assay. We also demonstrated that the addition of antibodies specific to either pembrolizumab or nivolumab could block detection of the drugs in the assay, thus providing a potential strategy to mitigate this cross-reactivity. In addition, we demonstrated that pembrolizumab, nivolumab, or anti-drug antibodies to either of these mAbs do not interfere in the cemiplimab bridging ADA assay. However, pembrolizumab and nivolumab generate a false-positive response in the target-capture competitive ligand-binding NAb assay. With the increasing number of clinical trials with different biotherapeutics that engage the same targets, we anticipate that cross-reactivity or interference in immunoassays from these biotherapeutics will be an ongoing bioanalytical challenge, in assays that use target-capture and generic anti-IgG detection reagents.

## MATERIALS AND METHODS

### Materials and Reagents

Cemiplimab (Libtayo®), the recombinant human PD-1 (extracellular domain), the monoclonal anti-cemiplimab antibody (ADA positive control), the neutralizing monoclonal anti-cemiplimab antibody (NAb positive control), the blocking anti-cemiplimab idiotypic antibody, and the anti-human IgG4 monoclonal antibody, were produced by Regeneron Pharmaceuticals, Inc. (Tarrytown, NY). Labeling of antibodies and targets with biotin using EZ-link Sulfo-NHS-LC-Biotin (Thermo Fisher Scientific), and with ruthenium NHS ester (MSD), was performed according to the manufacturer’s instructions.

Anti-nivolumab and anti-pembrolizumab monoclonal antibodies were purchased from BioRad (Hercules, CA). Pembrolizumab (Keytruda®) was manufactured by Merck Sharp & Dohme Corp. (Kenilworth, NJ). Nivolumab (Opdivo®) was manufactured by Bristol Myer Squibb (New York, NY).

All solutions, unless otherwise specified, were prepared in assay buffer (1% BSA in 1X PBS). Read Buffer T (4X) and the Streptavidin-coated microplates were purchased from Meso Scale Discovery (MSD, Gaithersburg, Maryland). Glacial acetic acid (17.4 M), NeutrAvidin-HRP, SuperSignal ELISA Pico Chemiluminescent Substrate, and 96-well microplates were purchased from Thermo Fisher Scientific (Waltham, MA). Trizma base (1.5 M) was purchased from Sigma (St Louis, MO). Wash solution and 10% BSA were purchased from SeraCare, (Milford, MA). Human serum, including that which was used for the negative quality controls (NQCs), was purchased from BioIVT (Westbury, NY).

### Immunoassay Procedures

All incubations noted below were performed at room temperature (RT) unless otherwise specified.

#### Drug Concentration Assay

Microplates were coated overnight at 4 °C with recombinant human PD-1 (0.5 µg/mL) and blocked with 5% (w/v) BSA for a minimum of 1 h. After blocking, human serum (2%) samples containing the indicated proteins were added to the microplates and incubated for 1 h. Subsequently, microplates were incubated with 100 ng/mL biotinylated mouse anti-human IgG4 mAb for 1 h, followed by incubation with 100 ng/mL NeutrAvidin-HRP for 1 h, and finally incubated with SuperSignal ELISA Pico Chemiluminescent Substrate, prepared according to manufacturer’s instructions, for 10 to 30 min. Microplates were read on a luminescence reader (BioTek, Winooski, VT).

#### Bridging Immunogenicity Assay

Unless otherwise specified, serum samples were diluted tenfold in 300 mM acetic acid and incubated for 30 min. Biotin and ruthenium labeled cemiplimab (2 µg/mL) were prepared in assay buffer containing 150 mM Tris (unless otherwise specified), and acid-treated serum samples were further diluted in the labeled reagent solution. After incubation for 1 h, samples were transferred to blocked (5% BSA) Streptavidin-coated plates and incubated for 1 h, before addition of Read Buffer and analysis on a QuickPlex SQ 120 reader (MSD, Gaithersburg, Maryland).

#### Target-Capture NAb Assay

Microplates were coated with recombinant human PD-1 (0.5 µg/mL) and blocked with 5% (w/v) BSA. Unless otherwise specified, serum samples were diluted tenfold in 300 mM acetic acid and incubated for a minimum of 10 min and then neutralized using a capture reagent solution containing 250 mM Tris, 20 ng/mL biotinylated-cemiplimab, and 5% BSA for 1 h. This was followed by incubation of 100 ng/mL Neutravidin-HRP for 1 h, and finally incubated with SuperSignal ELISA Pico Chemiluminescent Substrate, prepared according to manufacturer’s instructions, for 10 min. Microplates were read on a luminescence reader (Biotek, Winooski, VT).

### Clinical Sample Collection

Clinical serum samples were obtained from Phase 1 oncology studies. Studies included patients with documented anti-PD-1 medication use prior to enrollment in the trials with cemiplimab. The serum samples were collected from patients prior to dosing of study drug at baseline.

## RESULTS

### Cross-reactivity of Anti-PD-1 Antibodies in the Cemiplimab Target-Capture Drug Concentration Assay

**Fig. 1:**
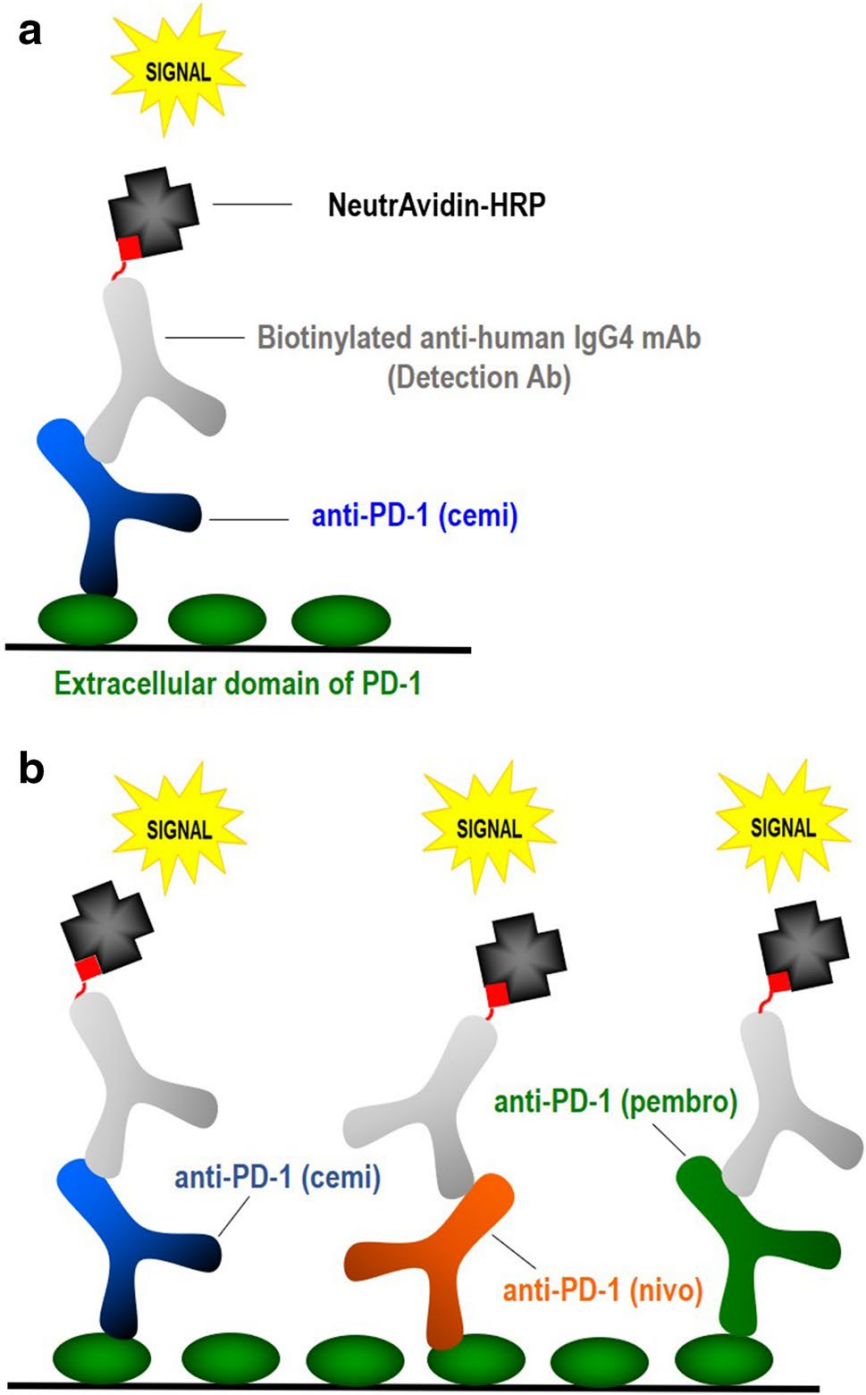
Illustrations of the functional cemiplimab drug concentration assay and cross-reactivity in serum. **a** Cemiplimab (cemi), captured on a PD-1 coated microplate is detected by a biotinylated anti-human IgG4 mAb followed by NeutrAvidin-HRP **b** Other anti-PD-1 human IgG4 mAbs such as nivolumab (nivo) or pembrolizumab (pembro) could also be detected

The cemiplimab enzyme-linked immunosorbent assay (ELISA) uses recombinant PD-1 as the capture reagent and a biotinylated anti-IgG4 mAb as the detection component (Fig. 1a). Other biologics that are also PD-1 specific and constructed with an IgG4 framework could potentially be detected in this target-capture method (Fig. 1b). Therefore, we set out to establish whether drug detected at baseline in clinical study samples was due to cross-reactivity from other IgG4 anti-PD-1 therapeutics.

**Fig. 2:**
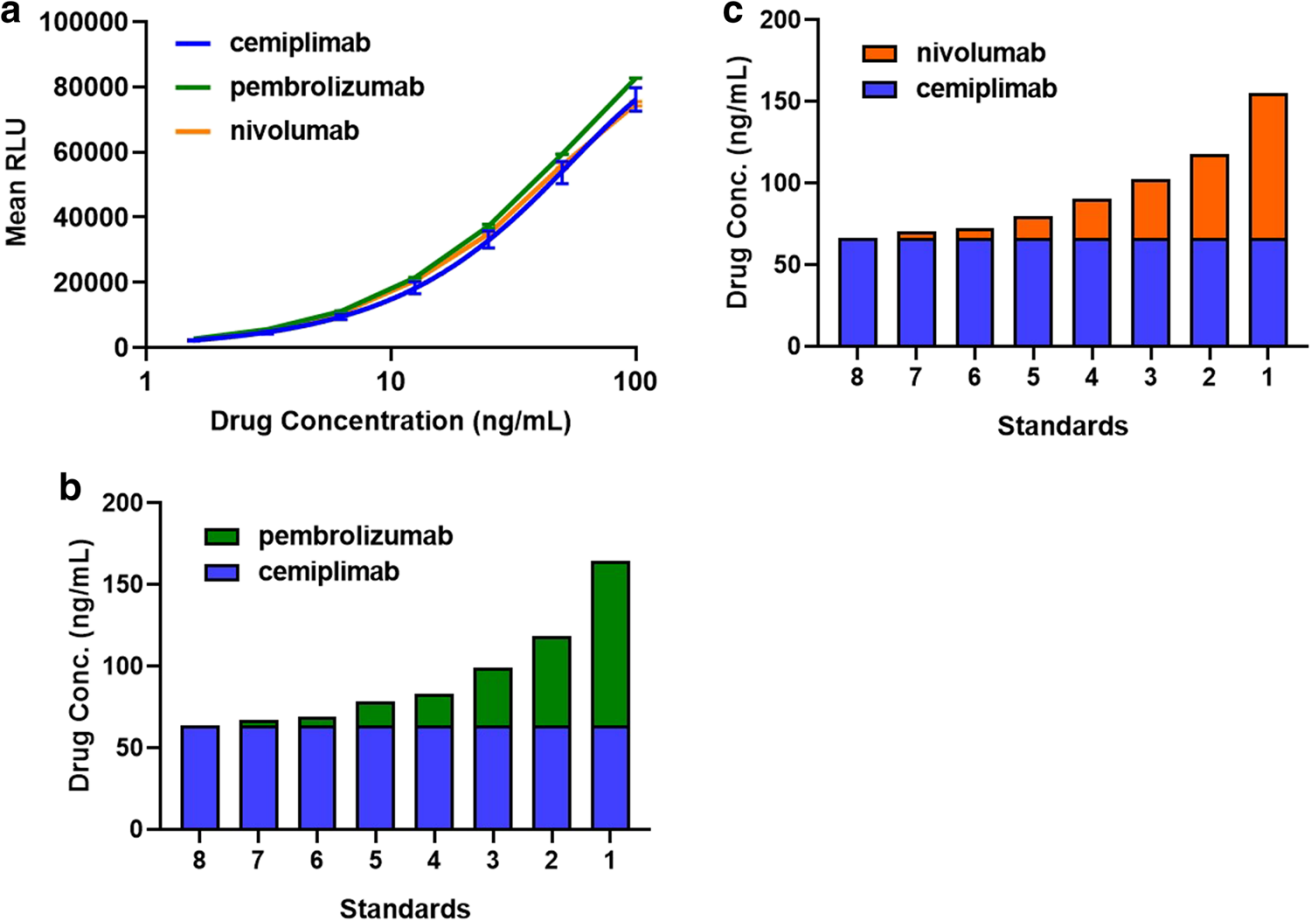
Other human IgG4 anti-PD-1 mAbs can be detected and accurately quantified in the functional cemiplimab drug concentration assay in serum. **a** Quantitation of serial dilutions (100 to 1.56 ng/mL) of cemiplimab (blue), pembrolizumab (green), and nivolumab (orange) in the assay. **b** Drug concentrations of HQC samples spiked with serial dilutions of pembrolizumab, interpolated from the cemiplimab standard curve, in the cemiplimab drug concentration ELISA **c** Drug concentrations of HQC samples spiked with serial dilutions of nivolumab, interpolated from the cemiplimab standard curve, in the cemiplimab drug concentration assay

To determine whether other anti-PD-1 mAbs cross-react in the assay, twofold serial dilutions of each of the three mAbs (cemiplimab, pembrolizumab, and nivolumab) were prepared in human serum at concentrations of 5 to 0.078 µg/mL (100 to 1.56 ng/mL after minimum required dilution) and analyzed in the assay. The signal generated from the serial dilutions of all three mAbs was very similar, indicating that they can all be detected in the assay. Furthermore, analyte recovery of pembrolizumab and nivolumab concentrations when interpolated from the cemiplimab standard curve generated values within 20% of the nominal values, thus demonstrating that the mAbs can be accurately quantified in this assay (Fig. 2a).

To determine if there was an additive effect of pembrolizumab or nivolumab in the cemiplimab ELISA, cemiplimab at the high quality control level (HQC; 75 ng/mL) or the lower level of quantification (LLOQ; 1.56 ng/mL) was added to the serial dilutions of pembrolizumab and nivolumab (Fig. 2b and c). When interpolated off the cemiplimab standard curve, the concentration of detected drug was equal to the sum of pembrolizumab or nivolumab plus the HQC (Fig. 2b and c) or LLOQ (data not shown) level of cemiplimab. This indicated that within the quantitative range of the assay, all anti-PD-1 mAbs present in the sample would be detected and accurately quantified with similar sensitivity.

### Anti-idiotypic Blocking Antibodies Mitigate Cross-reactivity in the Drug Concentration Immunoassay in Spiked Samples and in Baseline Clinical Trial Samples

**Fig. 3:**
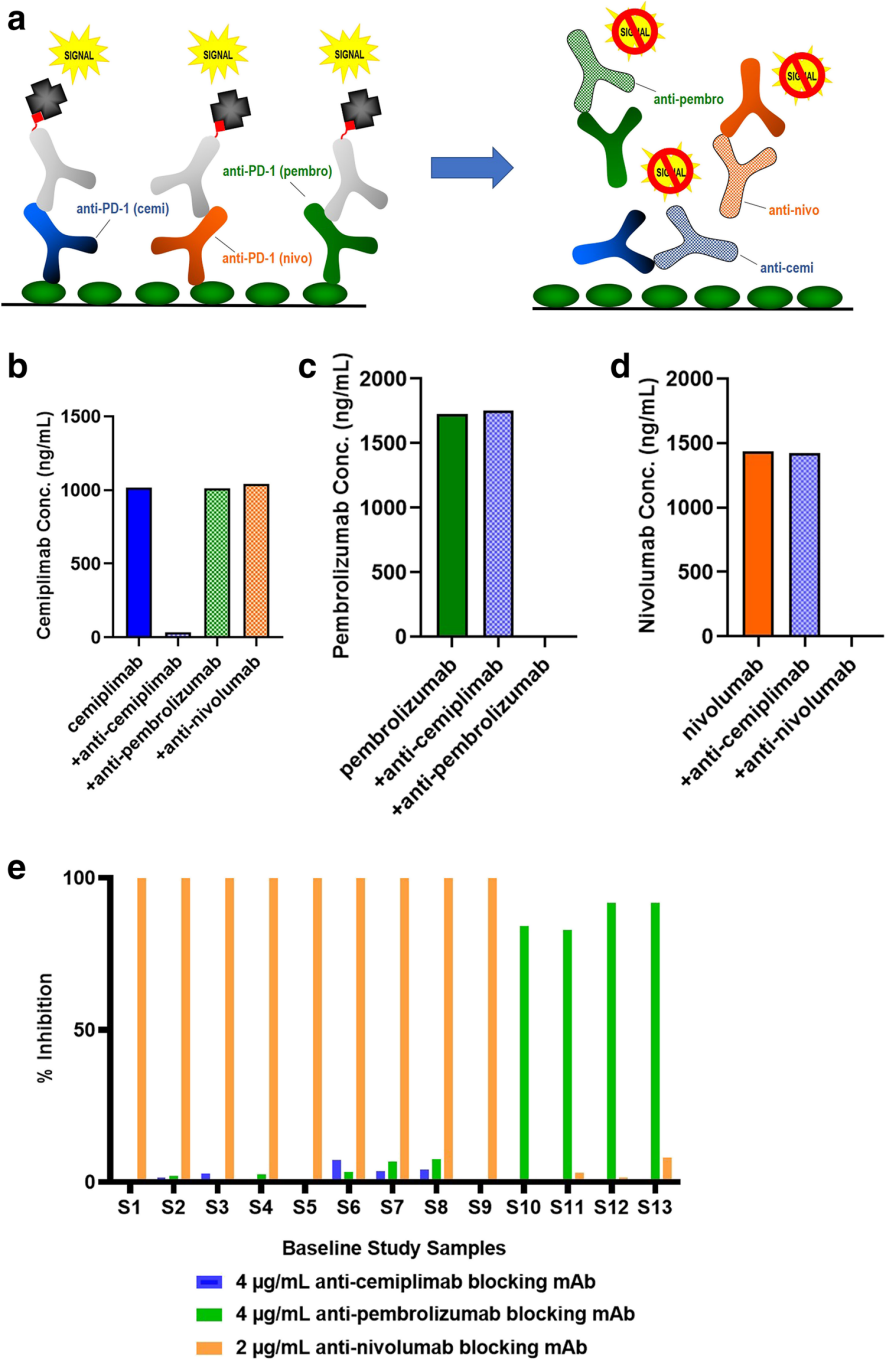
Anti-idiotypic antibodies block binding of anti-PD-1 mAbs in the cemiplimab target-capture drug concentration ELISA. **a** Schematic depicting the anti-idiotypic antibodies (checkerboard pattern) blocking each drug (solid colors) from binding to the PD-1 capture in the cemiplimab ELISA. **b** Mock sample serum control prepared with cemiplimab at the MQC concentration and tested in the presence or absence of 100 × concentration of all three anti-idiotypic antibodies in the cemiplimab ELISA. **c** Mock sample serum control prepared with pembrolizumab and tested in the presence of 100X concentration of the anti-pembrolizumab and the anti-cemiplimab antibodies in the cemiplimab ELISA. **d** Mock sample serum control prepared with nivolumab and tested in the presence of 100 × concentration of the anti-nivolumab and the anti-cemiplimab antibodies in the cemiplimab ELISA. **e** Baseline clinical samples with detectable responses in the cemiplimab ELISA from patients with prior exposure to pembrolizumab or nivolumab were evaluated in the presence of each of the three anti-idiotypic antibodies to demonstrate specific signal inhibition

A potential strategy to minimize cross-reactivity of pembrolizumab and nivolumab in the cemiplimab ELISA is to use anti-idiotypic antibodies to block binding of the other therapeutic mAbs to PD-1 on the plate (Fig. 3a). To test this strategy, mock serum samples were created by spiking serum with cemiplimab, pembrolizumab, and nivolumab at the middle quality control level (MQC; 1 µg/mL). The mock samples were then tested in the presence or absence of anti-cemiplimab, anti-pembrolizumab, and anti-nivolumab antibodies at 100 × (100 µg/mL) the MQC concentration.

The results demonstrate the anti-idiotypic blocking antibodies specifically inhibited binding of the corresponding drug to PD-1 on the plate diminishing detection in the cemiplimab ELISA (Fig. 3b through d). The anti-idiotypic antibodies did not cross-react or interfere with quantification of the other mAbs in the assay.

This strategy could also be used to confirm the identity of any anti-PD-1 mAb in baseline clinical samples from patients previously treated with an anti-PD-1 mAb. To evaluate this approach, baseline samples collected from patients with prior anti-PD-1 exposure to either pembrolizumab or nivolumab were analyzed in the presence and absence of each of the three anti-idiotypic antibodies (Fig. 3e). In every sample, assay signal was markedly inhibited (greater than 80%) by only the anti-idiotypic antibody that corresponded to each patient’s anti-PD-1 medication history (Fig. 3e).

### Potential Impact of Other Anti-PD-1 Biologics on the Specificity and Selectivity of the Cemiplimab ADA Assay

**Fig. 4:**
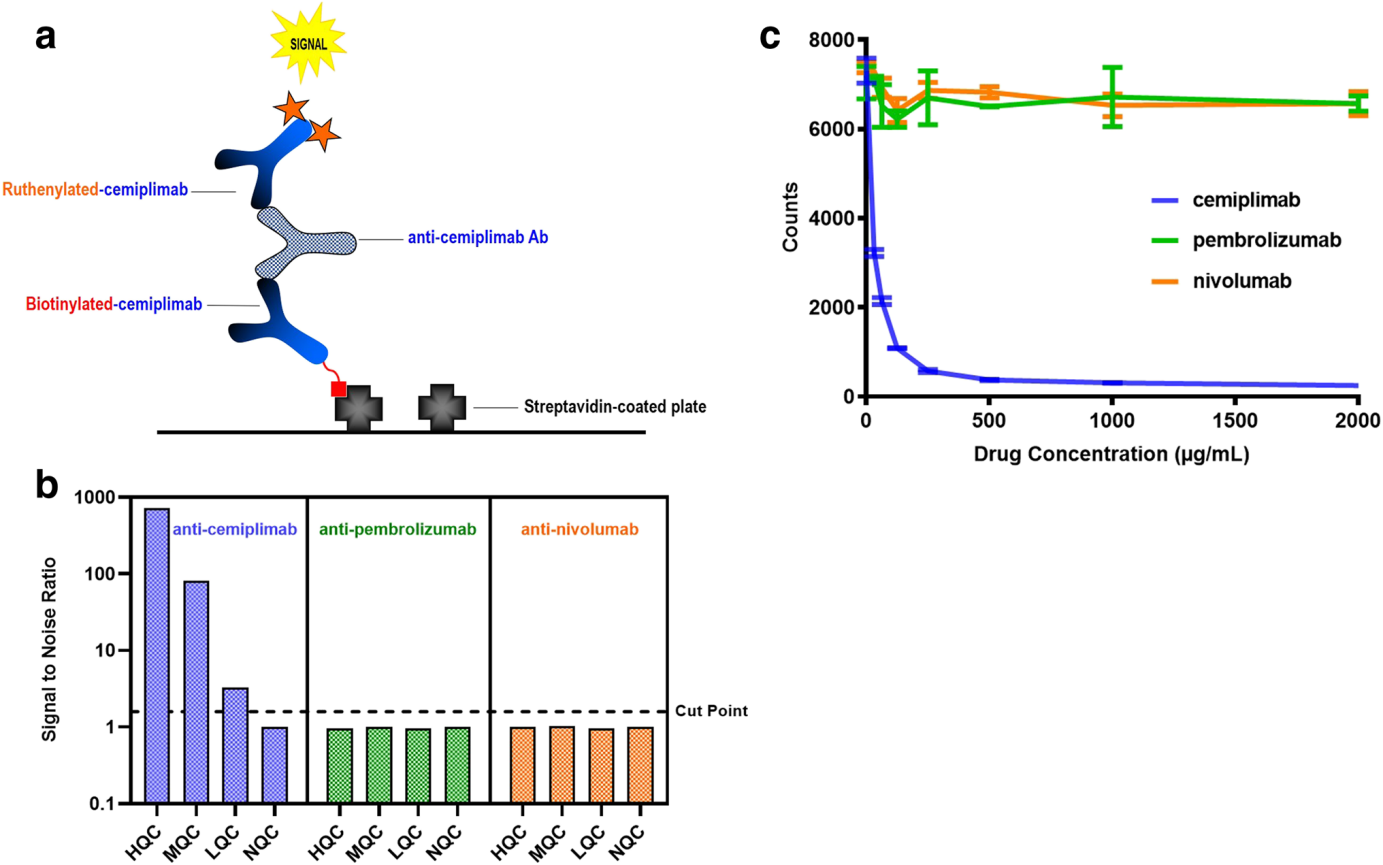
The cemiplimab ADA assay is specific only for anti-cemiplimab antibodies, and other anti-PD-1 mAbs do not interfere in the method. **a** Schematic of the cemiplimab bridging ADA assay in which ADA in the samples bridge between biotin- and ruthenium-labeled cemiplimab generating signal in the method. **b** Signal-to-noise ratio in the ADA assay for control samples containing anti-cemiplimab, anti-nivolumab or anti-pembrolizumab antibodies at three concentrations (X, Y, and Z ng/mL) in serum. **c** Signal in the ADA assay of anti-cemiplimab antibody control samples (500 ng/mL) tested in the presence of serial dilutions of cemiplimab, pembrolizumab, and nivolumab at the indicated concentrations

Prior exposure to the same class of anti-PD-1 mAb raises the possibility that some patients previously treated with pembrolizumab or nivolumab may generate ADA that cross-react in the anti-cemiplimab ADA assay. The bridging cemiplimab ADA assay uses a mouse anti-cemiplimab antibody as the positive control and biotinylated-cemiplimab and ruthenium-labeled cemiplimab as bridge components (Fig. 4a).

Serum positive control samples were prepared containing specific anti-cemiplimab, anti-pembrolizumab, or anti-nivolumab antibodies and were analyzed in the ADA assay. Anti-cemiplimab positive control samples generated a strong signal in the assay, while the anti-pembrolizumab or anti-nivolumab samples generated signal approximately equivalent to the negative control samples (Fig. 4b). This suggests that anti-pembrolizumab or anti-nivolumab antibodies generated in patients treated with these drugs likely will not interfere with the detection of anti-cemiplimab antibodies.

To test whether residual concentrations of pembrolizumab or nivolumab in circulation can impact the detection of cemiplimab ADA, samples containing an anti-cemiplimab monoclonal antibody (500 ng/mL) were tested in the presence of increasing concentrations of either pembrolizumab or nivolumab. The highest concentration of each drug tested (2 mg/mL), was greater than the Cmax levels observed in the clinical study samples (12, 13). These results demonstrated that even at high concentrations of pembrolizumab or nivolumab, detection of the anti-cemiplimab antibody was not impacted by the presence of either antibody in serum (Fig. 4c).

As a control, cemiplimab was also spiked at high concentrations in the assay. As expected, this reduced the anti-cemiplimab antibody assay signal, although control samples (500 ng/mL) remained positive in the assay when spiked with cemiplimab at concentrations greater than 500 µg/mL, confirming the cemiplimab drug tolerance level of the assay (Fig. 4c). These experiments demonstrate that the cemiplimab ADA assay is specific only for anti-drug antibodies directed to the variable domain of cemiplimab and is not impacted by the presence of other anti-PD-1 mAbs.

Collectively, these results demonstrate the specificity and suitability of our cemiplimab ADA assay for the detection of anti-cemiplimab antibodies in the presence of other anti-PD-1 ADA or residual anti-PD-1 therapeutics.

### Interference from Other PD-1 Therapies on a Target-Capture NAb Assay

**Fig. 5:**
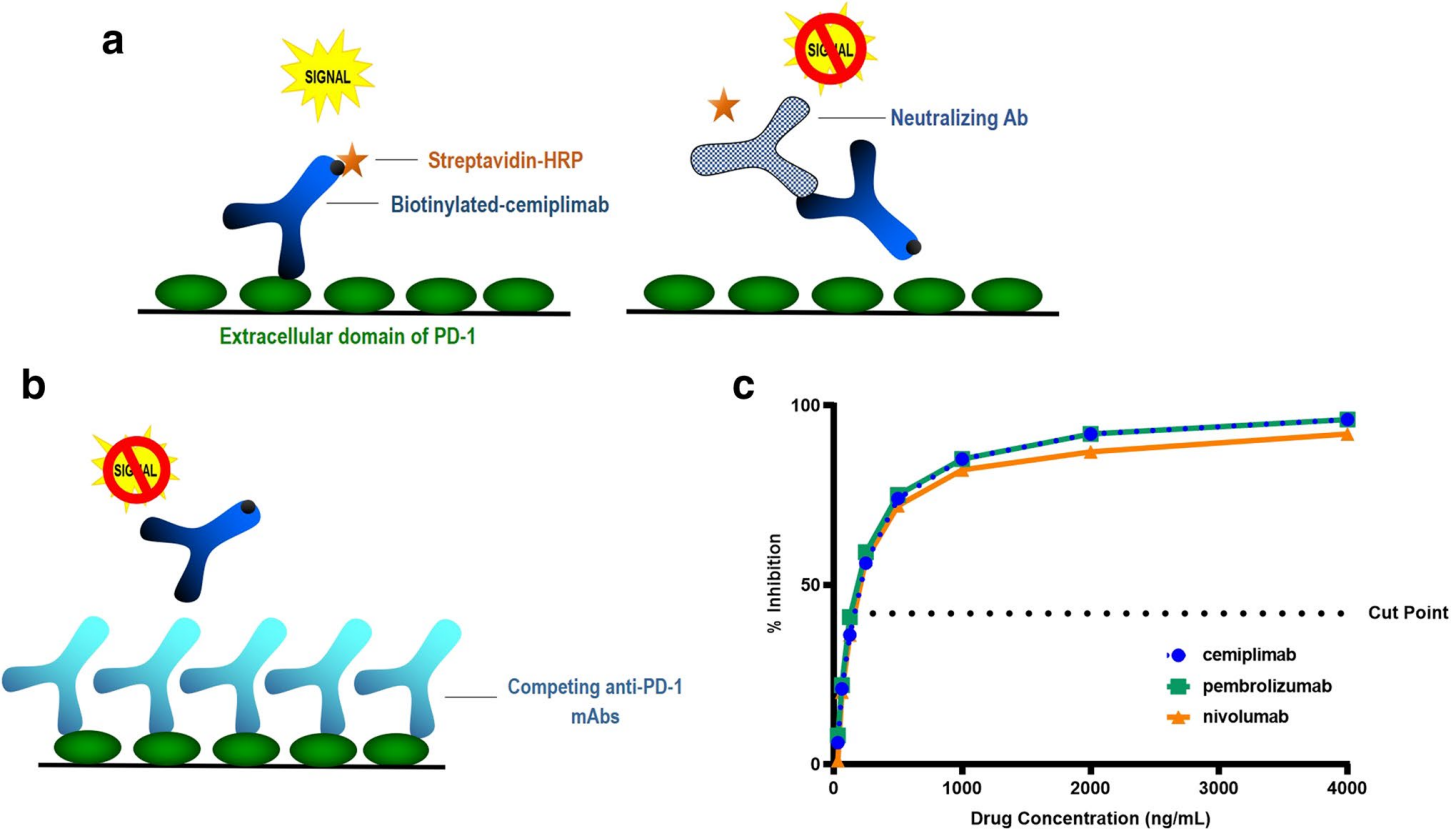
Anti-PD-1 biologics generate a false-positive response in a target-capture NAb assay in serum. **a** Schematic depicting the target-capture NAb assay. In the absence of NAb, biotinylated cemiplimab binds to a PD-1 coated plate, followed by streptavidin conjugated to HRP, generating signal in the assay. The presence of NAb inhibits biotin-cemiplimab binding to PD-1, resulting in signal reduction. **b** Schematic of a false-positive NAb response in the presence of anti-PD-1 mAbs that bind to the PD-1 coated plate preventing biotinylated-cemplimab from generating signal in the assay. **c** Assay signal inhibition (%Inhibition) in a cemiplimab target-capture NAb after addition of serially-diluted cemiplimab, nivolumab, or pembrolizumab, at concentrations ranging from 4000 to 31.25 ng/mL

Confirmed ADA positive samples were further assessed in a NAb assay to evaluate the ability to neutralize the biological activity of the drug. A competitive ligand-binding NAb assay was developed that uses recombinant PD-1 as the capture reagent and biotinylated-cemiplimab and streptavidin-HRP as the detection components (Fig. 5a). When present in a serum sample, NAbs will bind to biotinylated cemiplimab, preventing binding to the PD-1 coated microplate and inhibiting the assay signal (Fig. 5a). However, in this assay format, the presence of other anti-PD-1 biologics could also compete with biotinylated cemiplimab for PD-1 binding, potentially generating a false-positive NAb result (Fig. 5b).

To test this, cemiplimab, pembrolizumab, and nivolumab were serially diluted in serum from 4000 to 31.3 ng/mL and analyzed in the target-capture NAb assay. As demonstrated in Fig. 5c, a false-positive NAb signal was detected when approximately 155 ng/mL of any of these anti-PD-1 drugs were added to the competitive ligand-binding NAb assay, which is approximately 1000-fold lower than steady state drug concentrations (12–14). In contrast, excess cemiplimab (or other anti-PD-1 mAbs) do not generate false-positive responses in a drug-capture competitive ligand-binding NAb assay, as excess therapeutic from the previous capture step is washed away before addition of labeled target as detection reagent (not shown).

## DISCUSSION

There are numerous therapeutic targets for which multiple biologic drugs are approved, including products targeting TNF-α, PD-1, or PD-L1 (Table I) (15). Because these targets are clinically validated, many biotherapeutics are being investigated in novel combinations (or as biosimilars) for the treatment of new indications or to improve efficacy in existing populations. During development of these new drugs, in particular for oncology therapies in the PD-1 and PD-L1 classes, trial participants may transition to the investigational biotherapy (or combination) while still having detectable systemic concentrations of their prior therapy (10). These prior drugs have the potential to cross-react or interfere in bioanalytical assays for the new therapy, especially with target-capture-based methods.

In cemiplimab clinical studies, enrollment of patients who had received prior anti-PD-1 therapy was permitted in some cohorts. Baseline samples (taken prior to cemiplimab administration) for some of these patients had high drug levels in the cemiplimab drug concentration assay. We demonstrated that the assay was able to detect pembrolizumab or nivolumab in spiked samples, with approximately equivalent quantitation to cemiplimab. In addition, we were able to specifically inhibit assay signal by addition of anti-idiotype mAbs directed against each of the respective drugs. Using this approach, we identified patients that had previously received either pembrolizumab or nivolumab, which aligned with patient’s prior medication history.

In addition to investigating the functional cemiplimab drug concentration assay, we wanted to understand whether pre-existing pembrolizumab or nivolumab, or ADA directed against either pembrolizumab or nivolumab, could interfere in the cemiplimab immunogenicity assays. In the bridging ADA assay, the presence of pembrolizumab or nivolumab did not interfere with the detection of the anti-cemiplimab positive control. Furthermore, only antibodies specific for cemiplimab were detected in this assay, with no cross-reactivity from specific anti-pembrolizumab or anti-nivolumab antibodies. Although these results collectively confirm the specificity of our anti-cemiplimab ADA assay, it does not negate the possibility that ADA against other anti-PD-1 therapeutics may already exist but cannot be detected.

In the competitive ligand-binding NAb assay, neither pembrolizumab nor nivolumab interfered in the assay when configured in a drug-capture format (not shown), since these molecules were washed away before addition of the labeled target. However, in a target-capture format, the presence of either pembrolizumab or nivolumab generated a false-positive NAb response due to target binding and the resulting competition with the biotinylated-cemiplimab detection antibody, even at relatively low concentrations. Therefore, a drug-capture format is recommended for competitive ligand-binding NAb assays to avoid false-positive responses from other biotherapeutics to the same target (17).

It is unknown whether true treatment-emergent anti-pembrolizumab or anti-nivolumab immunogenicity correlates to subsequent ADA responses to other anti-PD-1 mAbs. However, ADA to fully human or humanized mAbs are predominantly directed to the variable domains, and ADA directed to the unique regions of one molecule would be unlikely to cross-react with the other molecules (8, 16, 18). Furthermore, immunogenicity to one mAb may not be predictive of an ADA response after subsequent exposure to a different mAb that binds to the same target.

## CONCLUSION

We demonstrated that prior exposure to anti-PD-1 mAbs, pembrolizumab and nivolumab, can be detected and quantitated in the cemiplimab drug concentration immunoassay. However, at steady state for the new therapy, these prior biotherapeutics would likely not be detected. In addition, we demonstrated that these prior biotherapeutics or anti-drug antibodies to either of these mAbs do not interfere in the cemiplimab bridging ADA assay. However, in a target-capture competitive ligand-binding cemiplimab NAb assay, pembrolizumab and nivolumab generated a false-positive response.

Immunoassays that use the drug target as a reagent in the assay, especially in the capture step, are potentially susceptible to interference or cross-reactivity with other biologics directed to the same target. Some of the most widely prescribed pharmaceutical products are biologics with the same target, most prominently drugs targeting TNF-α, PD-1, or PD-L1. In oncology particularly, there are additional targets (other than PD-1/PD-L1) that have more than one approved therapy, including CD20, EGFR, and HER-2 (Table I) (15). Cross-reactivity or interference in immunoassays from previous exposure to biotherapeutics of the same class is an ongoing bioanalytical challenge with molecules directed to these targets (17, 19). This work highlights the importance of understanding both patient medication history and assay format, to ensure the most appropriate bioanalytical strategies are implemented.

## Abbreviations

ADA: Anti-drug antibody
CPI: Check-point inhibitor
IO: Immuno-oncology
I&I: Immunology and inflammation
mAb: Monoclonal antibody
NAb: Neutralizing antibody
PC: Positive control
PD-1: Programmed cell death protein 1
PK: Pharmacokinetics
TE: Treatment emergent
